# Demethylation of the Coding Region Triggers the Activation of the Human Testis-Specific *PDHA2* Gene in Somatic Tissues

**DOI:** 10.1371/journal.pone.0038076

**Published:** 2012-06-01

**Authors:** Ana Pinheiro, Maria João Nunes, Inês Milagre, Elsa Rodrigues, Maria João Silva, Isabel Tavares de Almeida, Isabel Rivera

**Affiliations:** 1 Research Institute for Medicines and Pharmaceutical Sciences (iMed.UL), Lisbon, Portugal; 2 Department of Biochemistry and Human Biology, Faculty of Pharmacy, University of Lisbon, Lisbon, Portugal; Peking University Health Science Center, China

## Abstract

Human *PDHA2* is a testis-specific gene that codes for the E_1_α subunit of Pyruvate Dehydrogenase Complex (PDC), a crucial enzyme system in cell energy metabolism. Since activation of the *PDHA2* gene in somatic cells could be a new therapeutic approach for PDC deficiency, we aimed to identify the regulatory mechanisms underlying the human *PDHA2* gene expression. Functional deletion studies revealed that the −122 to −6 promoter region is indispensable for basal expression of this TATA-less promoter, and suggested a role of an epigenetic program in the control of *PDHA2* gene expression. Indeed, treatment of SH-SY5Y cells with the hypomethylating agent 5-Aza-2′-deoxycytidine (DAC) promoted the reactivation of the *PDHA2* gene, by inducing the recruitment of the RNA polymerase II to the proximal promoter region and the consequent increase in *PDHA2* mRNA levels. Bisulfite sequencing analysis revealed that DAC treatment induced a significant demethylation of the CpG island II (nucleotides +197 to +460) in *PDHA2* coding region, while the promoter region remained highly methylated. Taken together with our previous results that show an *in vivo* correlation between *PDHA2* expression and the demethylation of the CpG island II in testis germ cells, the present results show that internal methylation of the *PDHA2* gene plays a part in its repression in somatic cells. In conclusion, our data support the novel finding that methylation of the *PDHA2* coding region can inhibit gene transcription. This represents a key mechanism for absence of *PDHA2* expression in somatic cells and a target for PDC therapy.

## Introduction

Pyruvate dehydrogenase complex (PDC) is a mitochondrial matrix enzyme system that catalyses the oxidative decarboxylation of pyruvate to acetyl-CoA, a key metabolite for energy metabolism. The rate-limiting component is the E_1_ enzyme, which is a heterotetramer (α_2_β_2_). The α subunit, besides forming with the β subunit the active and the cofactor binding sites, is also the target for regulatory mechanisms governing global activity of PDC.

**Figure 1:**
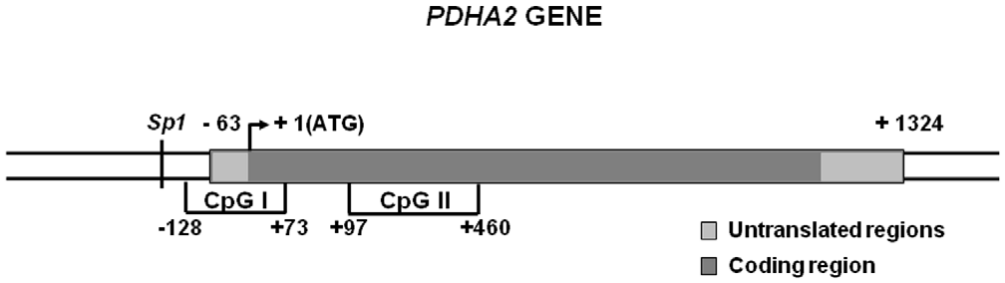
Schematic representation of the *PDHA2* gene showing the localization of the 2 CpG islands (CpG I and CpG II), the transcriptional start site and the putative Sp1 binding site.

The E_1_α subunit can be encoded by two different genes: *PDHA1* located on X chromosome and expressed in somatic tissues; and *PDHA2* an intronless gene located on chromosome 4 (Fig. 1). This autosomal gene is repressed in all somatic tissues but actively transcribed in post-meiotic germ cells where the X chromosome is absent or inactive [1], [2]. It was suggested that the translocation of *PDHA* to the eutherian X chromosome, which is inactivated during spermatogenesis, led to the evolution of this second testis-specific *locus* by retroposition to an autosome [3].

Besides the potential of *PDHA2* as a model for unraveling the mechanisms that govern gene expression during spermatogenesis [4], some authors also postulated that *PDHA2* gene activation in somatic cells could be an effective therapy for PDC deficiency [5], an inborn error of metabolism mainly caused by mutations in *PDHA1* gene [6].

The amount of published work on *PDHA2* gene regulation is scarce and the studies performed relied on the mouse orthologue, *Pdha2* [4], [7], [8], [9], [10]. However, it is believed that the regulatory mechanisms that control these orthologous genes are different, once there is no gross homology between their promoters, which seem to have evolved from different retroposons [11].

Datta and collaborators publish the first and, to our knowledge, unique study on human *PDHA2* gene regulation [5]. They isolated and characterized approximately 800 nucleotides of the *PDHA2* promoter region, identified the location of the transcription start site and performed functional studies that suggested the existence of multiple regulatory elements. More importantly, these authors proposed that the *PDHA2* tissue-specific expression could be modulated by mechanisms, such as DNA methylation, that would limit *PDHA2* expression to spermatogenic cells.

Indeed, DNA methylation is a widely known epigenetic mechanism that plays a central role in the selective expression of particular genes in different tissues. The methylation of cytosine residues acts as a negative regulator of transcription by three potential mechanisms: direct interference with the binding of specific transcription factors to promoters, direct binding of specific transcriptional repressors and alteration of the chromatin structure [12]. DNA methylation can be inhibited by 5-Aza-2′-deoxycytidine (DAC), a potent inhibitor of DNA methyltransferase (DNMT) activity, through the irreversible binding of DNMTs to DAC substituted DNA [13].

Recently, we have demonstrated an *in vivo* correlation between an increase in *PDHA2* mRNA levels and the demethylation of a CpG island in its coding region [14], which is a strong evidence for a role of DNA methylation in the epigenetic control of the human *PDHA2* gene. In this study we show that inhibition of DNMTs with DAC reactivates *PDHA2* gene expression in SH-SY5Y cells, in a demethylation-dependent manner.

## Results

### *PDHA2* promoter constructs display basal activity in human somatic cell lines

Human *PDHA2* gene expression is restricted to post-meiotic germ cells [2] suggesting the absence of positive modulating factors and/or the presence of repressors in somatic tissues. However, the lack of suitable human spermatogenic germ cell lines makes it difficult to easily elucidate the regulatory mechanisms involved upon *PDHA2* expression.

**Figure 2:**
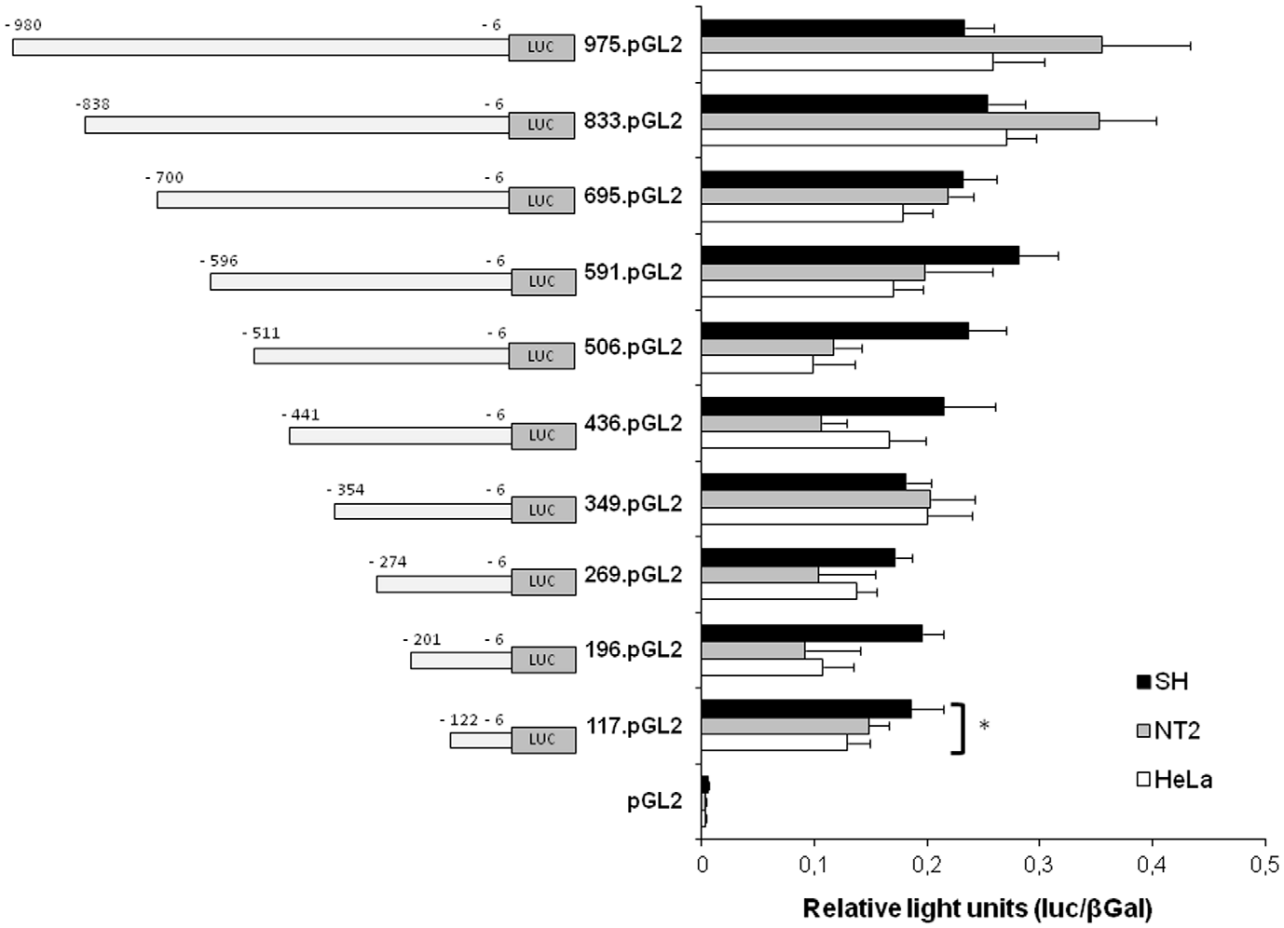
Functional deletion analysis of the human *PDHA2* gene promoter. Progressive 5′ deletion constructs were transiently transfected into HeLa, NT2 and SH-SY5Y cell lines. Transfections were carried out using 0.5 µg of the *PDHA2* reporter constructs or the empty pGL2 vector. Normalized luciferase activities were expressed as mean values ± SEM of duplicates for a minimum of three experiments (*p<0.001).

In order to evaluate the regions important for transcriptional modulation and, more precisely, to define the regions putatively involved in the repression of *PDHA2* gene in somatic cells, several deletion promoter constructs were generated (Fig. 2), and their ability to direct expression of the reporter luciferase gene was analyzed after transient transfection in different somatic cell lines. Accordingly, the various *PDHA2* reporter plasmids and the parental pGL2-Basic vector were transfected into HeLa (cervix adenocarcinoma), NT2 (human teratocarcinoma) and SH-SY5Y (human neuroblastoma) cells, which do not normally express *PDHA2* mRNA.

Surprisingly, *PDHA2* reporter constructs displayed high luciferase activities in all three somatic cell lines (Fig. 2). Significant differences were found between the luciferase activities of the several constructs, in HeLa (ANOVA one-way test: F = 20.29, df = 10, p<0.001), NT2 (ANOVA one-way test: F = 26.01, df = 10, p<0.001), and SH-SY5Y (ANOVA one-way test: F = 48.89, df = 10, p<0.001) cells. However, *post hoc* comparisons only revealed significant differences between the luciferase activities of the empty pGL2-Basic and all the other reporter constructs (Tukey HSD p<0.001).

These results firstly show that the region between nucleotides −122 and −6 seems to contain all the sequences necessary to drive transcription initiation, and thus most probably corresponding to the basal proximal promoter. Secondly, the fact that *PDHA2* promoter reporter constructs present high luciferase activity in somatic cell lines where the *PDHA2* mRNA cannot be detected, and that there are no significant differences between the luciferase activities of the different deletion constructs, suggest an important role of epigenetic modifications in the regulation *PDHA2* tissue-specific expression.

### *In vitro* methylation prevents *PDHA2* promoter constructs activity

**Figure 3:**
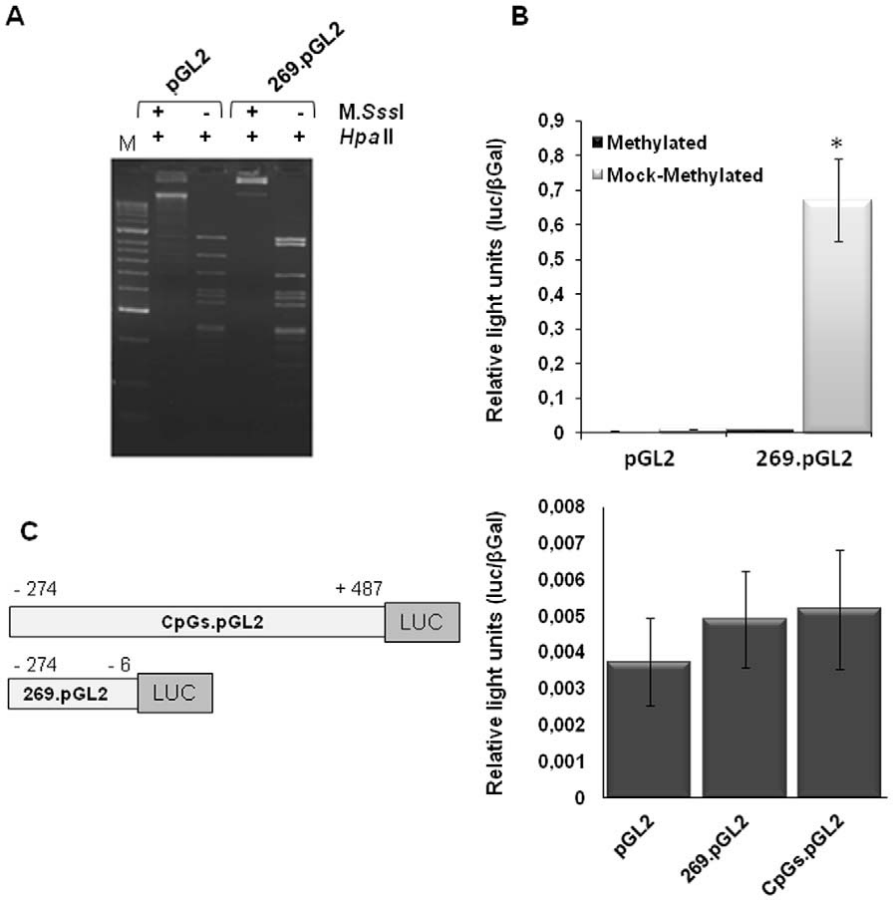
*In vitro* methylation with SssI methylase abrogates *PDHA2* promoter constructs activity. Constructs, either methylated or mock-methylated, were transiently transfected into SH-SY5Y cell line. Transfections were carried out using 0.5 µg of the *PDHA2* reporter constructs or the empty pGL2 vector. Normalized luciferase activities were expressed as mean values ± SEM of duplicates for a minimum of three experiments (*p<0.001). (A) Control of the methylation reaction by digestion with the methylation-sensitive endonuclease HpaII. (B) Methylation of *PDHA2* constructs prevents proximal promoter activity. (C) No significant decrease in the luciferase activity was observed in the *PDHA2* construct including the CpG II region (CpGs.pGL2).

To evaluate the role of DNA methylation on the activity of the *PDHA2* proximal promoter, we performed an *in vitro* methylation assay using *Sss*I methylase (Fig. 3A). The results revealed that the *PDHA2* proximal promoter activity was completely abolished after *in vitro* methylation (Fig. 3B). Moreover, since we had previously demonstrated an *in vivo* inverted correlation between methylation of a CpG island localized in the coding region (see CpG II in Fig. 1) and the *PDHA2* mRNA levels [14], we wanted to assess if the presence of a methylated CpG island II region could further decrease *PDHA2* construct's luciferase activity. Nevertheless, since there were already no significant differences between the luciferase activity of the 296.pGL2 construct and of the empty pGL2.Basic vector (Fig. 3B and 3C), the inclusion of the CpG II region did not result in a further decrease in promoter activity.

These results indeed show that methylation plays an important repressive role upon *PDHA2* gene expression.

### Activation of *PDHA2* expression in somatic cells after treatment with epigenetic drugs

To further investigate the role of epigenetic mechanisms upon *PDHA2* gene regulation, we treated SH-SY5Y cells, where this gene is not normally expressed, with a potent inhibitor of the *de novo* methylation (DAC) and with a pharmacological inhibitor of histone deacetylases (TSA).

**Figure 4:**
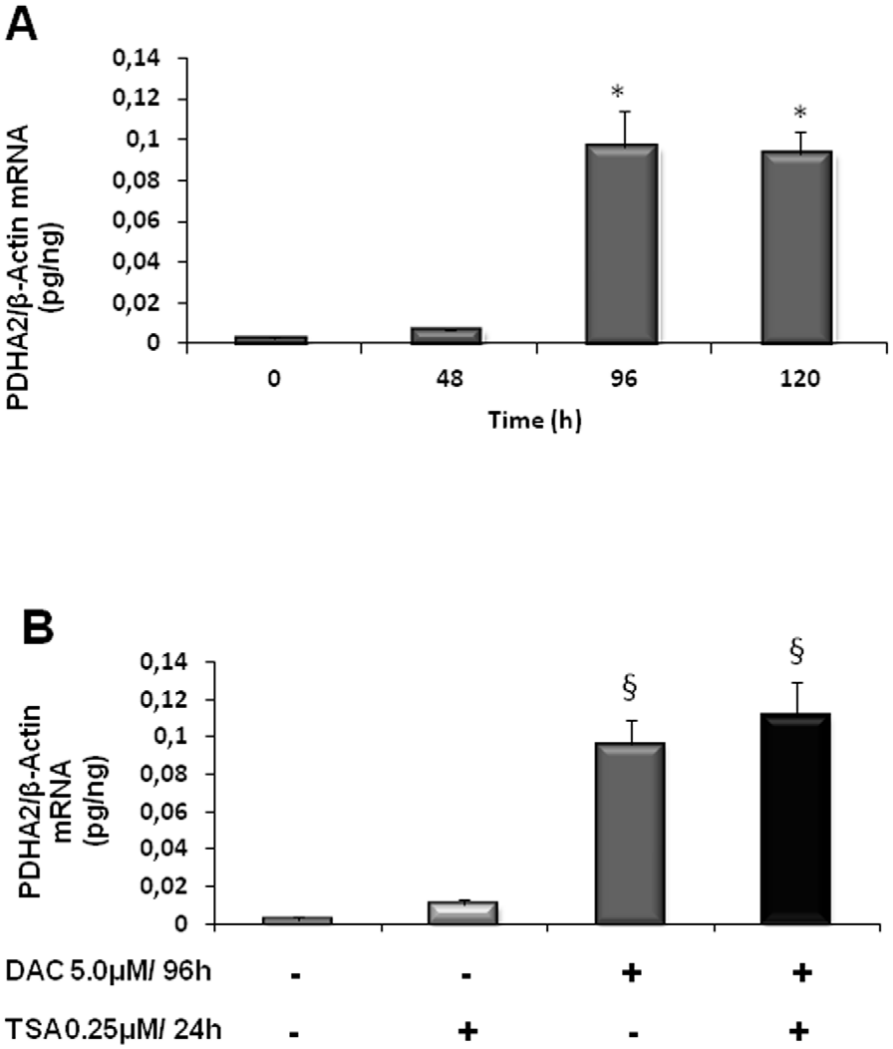
DAC increases *PDHA2* mRNA levels in SH-SY5Y cells. Real-time PCR analysis of *PDHA2* steady-state mRNA transcript levels in SH-SY5Y cells treated with 5 µM DAC for the indicated time points (A), and with 5 µM DAC for 96 h and/or 0.25 µM TSA for 24 h (B). Values were normalized to the internal standard β-actin. Data represent means ± SEM of at least three independent experiments and was expressed as pg of *PDHA2* mRNA per ng of β-actin mRNA (* p<0.001; § p<0.01).

*PDHA2* expression was quantified by real-time PCR (qPCR) and the results revealed that after treatment with 5 µM DAC for 96 h or 120 h, there is a significant increase of *PDHA2* mRNA, when compared with untreated cells (ANOVA one-way test: F = 27.84, df = 3, p<0.001; Tukey HSD for unequal N p<0.001) (Fig. 4A).

Since it is generally accepted that DAC and TSA synergistically affect gene expression, by a mechanism that depends on promoter demethylation induced by DAC and subsequent reinforcement by histone acetylation induced by TSA, we also treated SH-SY5Y with TSA for 24 h, and with DAC for 96 h prior to a 24 h treatment with TSA. Incubation with 0.25 µM TSA for 24 h did not significantly affect *PDHA2* mRNA levels. Moreover, pretreatment with DAC for 96 h, prior to the 24 h TSA treatment, did not further increase the observed *PDHA2* mRNA accumulation with DAC treatment only (Fig. 4B).

**Figure 5:**
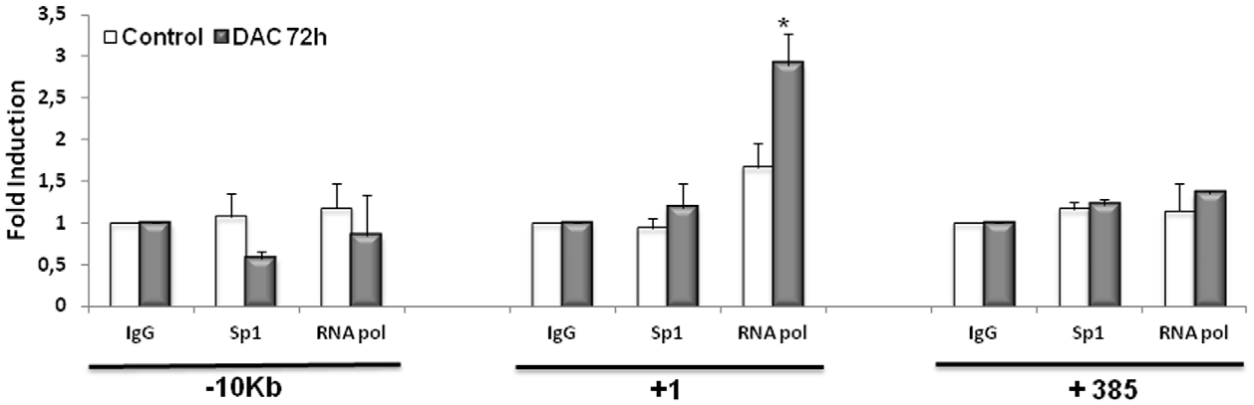
Recruitment of RNA pol II to the human *PDHA2* gene. Chromatin from SH-SY5Y was prepared at 72 hours after treatment with 5 µM DAC and precipitated with antibodies directed against IgG, Sp1 and RNA pol II. After DNA recovery, the precipitates were evaluated by real-time PCR as described in Experimental Procedures. Results are expressed as fold change over IgG and represent means of at least three independent experiments ± SEM (* p<0.001).

In order to corroborate the fact that DAC treatment induces a derepression of *PDHA2* gene transcription, we analyzed the recruitment of RNA polymerase II (RNA pol II) to this gene by chromatin immunoprecipitation. We have designed three sets of primers, one targeting the promoter region (+1 bp), another targeting a distal upstream region (−10 kb) and a third one targeting the CpG island II (+385 pb). Our results showed that we could only detect RNA pol II binding to the +1 bp region after DAC treatment (Student's *t*-test, p<0.001), which most likely triggers the observed increase in *PDHA2* gene transcription (Fig. 5). Interestingly, we could not detect the occupancy of the putative Sp binding site, by the Sp1 transcription factor (Fig. 5), as previously suggested by Datta and co-workers [5]. Furthermore, we investigated the possible recruitment of the elongation marker H3K36me3 to the gene body, but the results showed no occupancy of the targeted region.

These data demonstrate that *PDHA2* gene transcription was reactivated by DAC treatment in SH-SY5Y cells, suggesting that DNA demethylation may play a pivotal role in *PDHA2* tissue-specific expression.

### DAC induces demethylation of *PDHA2* coding region

In order to confirm that DAC can induce the reactivation of *PDHA2* expression in SH-SY5Y cells, in a demethylation dependent manner, we analyzed the methylation status of *PDHA2* gene body by sodium bisulfite sequencing analysis.

**Figure 6:**
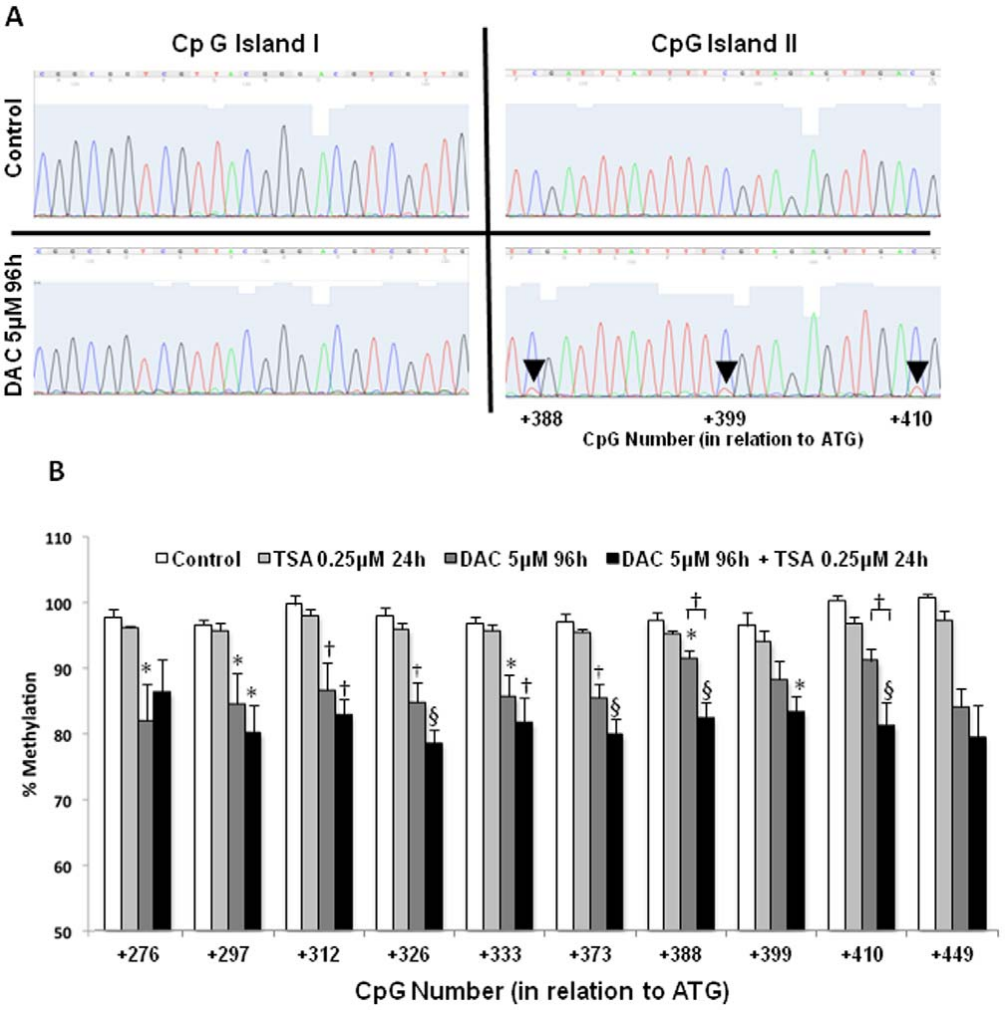
DNA methylation status of the human *PDHA2* gene after sodium bisulfite PCR sequencing. A) Sample chromatograms obtained for both *PDHA2* CpG Islands, before and after treatment with 5 µM DAC; B) DNA methylation status of CpG sites in the CpG island II located in the coding region of *PDHA2* gene, before and after treatment with 5 µM DAC for 96 h and/or 0.25 µM TSA for 24 h. Results represent means of at least three independent experiments ± SEM (* p<0.05; † *p*<0.01; § p<0.001 by Tukey HSD for unequal N test).

Since we have previously shown that the *PDHA2* gene contains two CpG islands, one located in the promoter region (nucleotides −128 to +73) and the other located in the coding region (nucleotides +197 to + 460) [14], we have analyzed the methylation patterns of both CpG islands. In accordance with our previous results, we observed that the CpG island I, located in the promoter region, is fully methylated in untreated SH-SY5Y cells, and seems to be insensitive to DAC, since it remains methylated after treatment with this drug (Fig. 6A). Effectively, its CpG sites remained fully methylated not only after DAC treatment, but after TSA and DAC plus TSA treatment, as well. Interestingly, the analysis of the CpG island II, located in the coding region, showed significant demethylation after treatment with DAC (Table S1) (Fig. 6A and 6B); TSA alone had no effect, but after pre-treatment with DAC, the demethylation level of this exonic CpG island seems to be slightly increased, which is particularly significant in the CpG sites +410 and +492 (Tukey for unequal N, p<0.01) (Fig. 6B).

**Figure 7:**
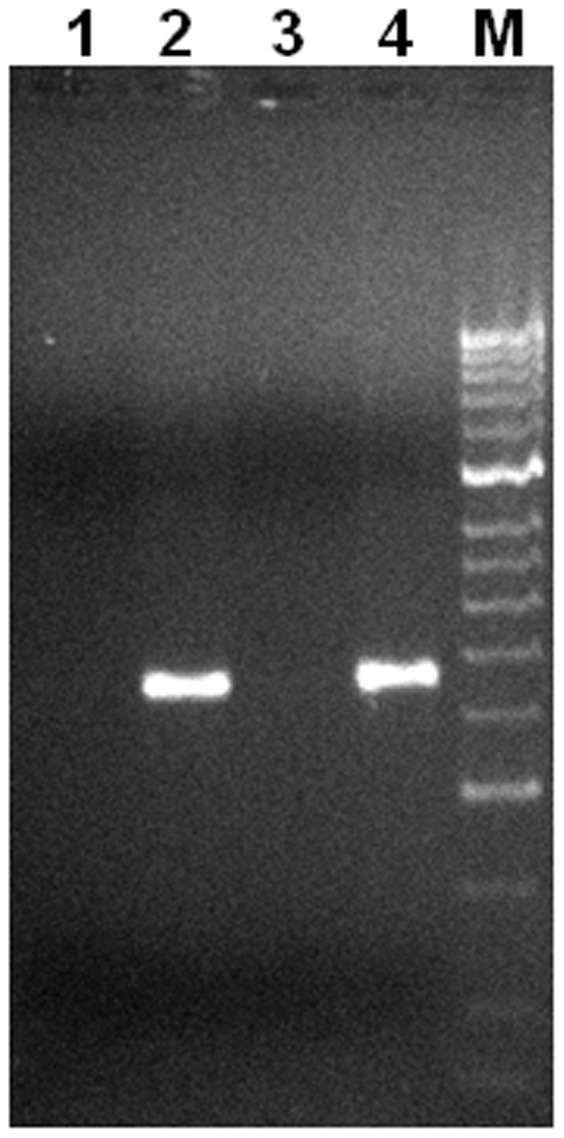
DAC activates a full-length *PDHA2* transcript. Conventional RT-PCR analysis, using primers hybridizing to the 5′ region, was used for detection of *PDHA2* transcripts. (1) SH-SY5Y cells before treatment. (2) SH-SY5Y cells after treatment with 5 µM DAC. (3) Negative control – no biological sample. (4) Positive control – mature spermatozoa sample. (M) HyperLadder II (Bioline, London, UK).

Recently, several potential alternative transcription start sites were identified in *PDHA2* gene [15]. These are located in the coding region encompassing the CpG island II. Therefore, one could hypothesize that demethylation of the *PDHA2* CpG island II corresponds to the demethylation of alternative promoters that control transcription of truncated *PDHA2* transcripts. Indeed, as the qPCR assay used a TaqMan probe hybridizing to the 3′UTR of the *PDHA2* mRNA, the presence of eventual shorter truncated transcripts could not be discarded. Accordingly, we further analyzed *PDHA2* expression by conventional RT-PCR with a set of specific primers designed to amplify the 5′ region of the full-length transcript (nucleotides −27 to +432). We detected the expected amplification product with 459 bp only in DAC-treated cells, which proves that DAC promotes the transcription of a full-length *PDHA2* transcript (Fig. 7).

These results are consistent with our previous work [14], and strongly suggest that *PDHA2* reactivation in somatic cells proceeds *via* a mechanism independent of promoter demethylation, most probably dependent on demethylation of the coding region.

## Discussion

*PDHA2* gene activation in somatic tissues has been postulated as a conceptual therapy for PDC deficiencies caused by *PDHA1* gene mutations [5], [16]. Accordingly, it is crucial to understand the mechanisms controlling human *PDHA2* tissue-specific expression, namely what factors are responsible for the silencing of this gene in somatic tissues, and its activation in spermatogenic ones (diploid and haploid germ cells). However, the lack of a suitable cell line to study the regulatory mechanisms underlying *PDHA2* gene expression has hampered this elucidation for a long time.

The first step towards understanding the regulation of a particular gene is the identification of regulatory elements and factors involved in basal expression. Accordingly, our studies began by the functional analysis of human *PDHA2* promoter. The results showed that *PDHA2* promoter-directed transcription of the luciferase reporter gene occurred in cultured somatic cells (HeLa, NT2 and SH-SY5Y), where *PDHA2* mRNA is undetectable. Deletion analysis further revealed that the region spanning from −122 to −6 is indispensable for basal expression of this TATA-less promoter. Moreover, and in each cell line, no significant differences were found among the reporter gene activities driven by all the other *PDHA2* deletion promoter constructs. Additionally, each construct displayed comparable reporter activities in the three different somatic cell lines.

These observations suggested that the mechanisms involved in the repression of *PDHA2* expression in somatic cells are not operative when the core promoter or the 5′ flanking region are transiently transfected into somatic cells. Moreover, these results corroborated the previous transactivation experiments described by Datta and colleagues [5], who also observed *PDHA2* promoter-directed transcription in human hepatocellular carcinoma cells. However, we could not replicate their results concerning the detection of several enhancer and repressor elements, because no significant differences in luciferase reporter activities were detected between the various deletion constructs. Additionally, the same authors suggested the existence of a putative Sp responsive element that would be important for Sp1-dependent transcription initiation [5]. However, our chromatin immunoprecipitation assays did not demonstrate any binding of Sp1 transcription factors to the proximal promoter of the *PDHA2* gene.

Additionally, and more importantly, our observation that *PDHA2* promoter reporter constructs presented high luciferase activity in all somatic cell lines, where the *PDHA2* mRNA cannot be detected, reinforces the idea that *PDHA2* tissue-specific expression may be under strict control of epigenetic mechanisms of regulation. Furthermore, *in vitro* methylation of *PDHA2* promoter constructs with *Sss*I methylase resulted in a complete abrogation of luciferase activity.

Our next approach was to further explore the involvement of epigenetics, namely DNA methylation and/or histone modifications, on the regulation of *PDHA2* gene expression. The results showed that treatment of SH-SY5Y cell cultures with DAC induced *PDHA2* derepression with a concomitant accumulation of *PDHA2* transcript. On the other hand, inhibition of histone deacetylation did not elicit any induction of *PDHA2* expression, nor did it potentiate the DAC effect.

Moreover, the accumulation of the *PDHA2* mRNA after DAC treatment was correlated with an enrichment of RNA pol II at the *PDHA2* proximal promoter (+1 bp region), which likely triggers the observed increase in *PDHA2* mRNA levels. The fact that treatment with DAC alone elicited a significant effect upon *PDHA2* gene expression suggests that changes associated with methylation are sufficient to drive transcription initiation of this testis-specific gene in somatic cells.

Once proved that DAC was able to induce *PDHA2* gene expression in cultured somatic cells, it would be important to investigate the methylation status of this gene, before and after treatment with the demethylating drug, in order to assure that demethylation was underlying *PDHA2* transcription. Our results demonstrated that DAC promoted a relevant demethylation of *PDHA2* coding region, which was fully methylated before treatment. However, the promoter CpG island I remained fully methylated, suggesting its insensibility to demethylation, at least by DAC treatment in the tested conditions. These findings corroborate our previous reported results, where we observed that *PDHA2* expressing tissues (i.e spermatogenic cells) presented the coding region completely demethylated, while non-expressing tissues displayed it fully methylated [14].

Moreover, these results also correlate with our recent finding concerning a family displaying *PDHA2* gene expression in somatic tissues [17], an interesting case that may configure the previously referred long dreamed therapy for PDC deficiency, i.e. the somatic activation of *PDHA2* expression. The results obtained either then by *in-vivo* or now by *ex-vivo* experiments are overlapping; actually, both methylation analyses revealed a correlation between demethylation of the coding region and *PDHA2* derepression. Moreover, and interestingly, the level of demethylation in the coding region is very similar in samples derived from the cultured cells treated with DAC and from the family individuals.

However, Yamashita and colleagues [15] recently identified multiple transcriptional start sites downstream of the canonical one (+248, +253 and +269), which are located precisely within the CpG island II region (nucleotides +197 to +460). Accordingly, this region could eventually harbor alternative promoter that would trigger the transcription of truncated *PDHA2* mRNAs. However, our RT-PCR results clearly show that DAC induces transcription of full-length transcripts. These data are corroborated by the significant enrichment of RNA pol II at the proximal promoter.

Taken together, our results confirm that methylation of the coding region is a key point in somatic cell silencing of *PDHA2* gene. Actually, DNA methylation appears to be particularly suitable for the regulation of germ line-specific genes, and this is probably related to the global demethylation process that occurs during the development of spermatogenic cells, which may provide the mechanism by which these germ line-specific genes are demethylated [18], [19]. And, despite the fact that DNA methylation does not seem to be the primary control mechanism regulating the programmed expression of most tissue-specific genes resulting in tissue differentiation, there are several examples that indicate that DNA methylation can serve as the primary control mechanism for the expression of a number of germ line-specific genes [20], [21], [22].

Although the expression of previously referred genes proceeds *via* a promoter methylation-dependent mechanism, we also can find in the literature some references to human genes that are regulated by methylation of the coding region, namely monocarboxylate transporter MCT3 [23]. Another particular interesting example is the mouse *Tact1/Actl7* gene [24], which is also intronless and testis-specific, like the *PDHA2* gene.

Indeed, it has already been described that methylation of the coding region, *per se*, can control gene expression by preventing promoter activity at the level of the chromatin structure. Indeed, CpG methylation induces a local repressive chromatin structure, mediated by the binding of methyl-CpG binding domain (MBD) proteins which recruit other proteins including sin3A and histone deacetylase; when a sufficient amount of CpGs is methylated, this repression is transmissible in *cis*, spreading for several hundred base pairs [25], [26], [27]. Accordingly, more or less distant methylated sequences, like the *PDHA2* coding region, can promote gene repression. Interestingly, and as stated by Nan and co-workers, this type of repression is greater if the promoter itself is methylated [28], which is the case of *PDHA2* promoter.

Furthermore, it has also been postulated that methylation of the coding region can inhibit gene expression by interfering with the elongation step rather than with transcription initiation, by causing RNA pol II to pause or to prematurely terminate [29], [30]. Moreover, the inhibitory effect upon elongation is prominent when methylation occurs near the start codon [31].

Based on this hypothesis, we attempted to explore elongation of *PDHA2* transcription by chromatin immunoprecipitation assays. The results did not show any particular differences in the chromatin recovered before and after DAC treatment, namely when using the elongation marker H3K36me3. A recent report has shown a clear correlation between H3K36me3 marking and transcriptional activity in intron-containing genes; however, in intronless genes H3K36me3 is detected at much lower levels irrespective of expression levels [32]. Indeed, there are other examples showing that transcriptional elongation is differently controlled in intronless genes when compared to longer intron-containing genes [33]. Accordingly, because *PDHA2* gene lacks introns, different approaches need to be designed.

In summary, we hypothesize that *PDHA2* gene belongs not only to a restricted group of germ line-specific genes that use DNA methylation as a primary silencing mechanism, but to a unique subset of those genes whose expression is regulated by the methylation status of the coding region. Furthermore, these new insights on the regulatory mechanism underlying *PDHA2* tissue-specific expression may open potential therapeutic avenues for PDC deficiency caused by *PDHA1* mutations.

## Materials and Methods

### Cloning the human *PDHA2* gene

Two different fragments of the human *PDHA2* gene (GenBank ID: M86808) were cloned in pCR®4Blunt-TOPO® vector (Invitrogen Corporation, Carlsbad, CA, USA) after PCR amplification with Platinum® *Pfx* Polymerase (Invitrogen) of genomic DNA isolated from circulating lymphocytes of a healthy individual. The first fragment harboring the *PDHA2* promoter region (nucleotides −980 to −6) was amplified using the primers pPDHA2-975 bp and pPDHA2-R listed in Table 1. The second fragment designed to harbor both CpG islands of *PDHA2* gene (−274 to +487) was amplified with pPDHA2-269 bp and PDHA2.CpGs-R primers (Table 1). The recombinant plasmids were sequenced by primer walking and named pPDHA2-TOPO and PDHA2.CpGs-TOPO, respectively. For sequence numbering, nucleotide +1 was assigned to the adenosine of the initiation translation codon ATG.

**Table 1:** Sequence of oligonucleotides used in the human *PDHA2* gene cloning.

Oligonucleotides	Sequence (5′→3′)
pPDHA2 – R	TCACGGAGTGCTGTAGATGGCTCGAGCCG
pPDHA2 – 975 bp	TGGAAACCTGCTGAAGACATT
pPDHA2 – 833 bp	AAGGAAAAGTGGAATGTCACAAA
pPDHA2 – 695 bp	ATACATTTTCCCTCCCCACT
pPDHA2 – 591 bp	GTTAACGTGCGTGTGCTTGT
pPDHA2 – 506 bp	GGCACATTATGGAGCAGGAT
pPDHA2 – 436 bp	TGGTAGGAAGAAATACCTTTGGA
pPDHA2 – 349 bp	TTGTCGGGAAAGCTTGAGAT
pPDHA2 – 269 bp	GCGATTAGGATGCCCTGTAG
pPDHA2 – 196 bp	GGCAGGCACTGTACAAATCA
PDHA2.CpGs – R	CGGCTCGAGCCATTGCCCCCATAGAAGT

### *PDHA2* promoter reporter constructs

Several different fragments derived from the human *PDHA2* promoter region were subcloned into the luciferase expression vector pGL2-Basic vector (Promega Corporation, Madison, WI USA). We used the pPDHA2-TOPO plasmid as a template to amplify fragments of different lengths, using different forward primers and a reverse primer, which contains a *XhoI* overhang site, listed in Table 1. The PCR products, amplified with the Platinum® *Pfx* Polymerase (Invitrogen), were subcloned in the pGL2 reporter plasmid into *SmaI/XhoI* sites generating plasmids 975.pGL2 (−980 to −6), 833.pGL2 (−838 to −6), 695.pGL2 (−700 to −6), 591.pGL2 (−596 to −6), 506.pGL2 (−511 to −6), 436.pGL2 (−441 to −6), 349.pGL2 (−354 to −6), 269.pGL2 (−274 to −6), and 196.pGL2 (−201 to −6). The pPDHA2-TOPO recombinant was also digested with enzymes *MslI/XhoI* and the 117 bp fragment was subcloned in the pGL2 reporter plasmid generating the 117.pGL2 recombinant (−122 to −6).

An ultimate *PDHA2* reporter construct was subcloned into the pGL2-Basic vector, using as template the PDHA2.CpGs-TOPO plasmid. This reporter construct was named CpGs.pGL2 and harbored both CpGs islands present in *PDHA2* gene.

### *In vitro* methylation of *PDHA2* constructs

M.SssI was used to methylate the CpG dinucleotides of the *PDHA2* gene/luciferase reporter constructs or the pGL2 basic vector, based on the protocol of the manufacturer (New England Biolabs, Beverly, MA). Mock reactions were carried out in parallel without adding the methylase. The samples were then incubated with methylation-sensitive restriction enzyme *Hpa*II, followed by agarose gel electrophoresis. Complete methylation of the CpG sites of the constructs was verified by protection of the methylated DNA from digestion by this enzyme. Methylated and mock-treated plasmids were purified with a QIAquick gel extraction kit (Qiagen, Hilden, Germany) and used for reporter gene analyses after transient transfection in somatic cell lines.

### Transactivation studies

The basal promoter activity of the different reporter plasmids was assayed by measuring the luciferase activity after transient transfection in HeLa, SH-SY5Y and Ntera2/clone D1 (NT2) cell lines.

To minimize variations in transfection efficiency, replicates were transfected in single batch suspension with FuGENE® HD (Roche Diagnostics GmbH, Penzberg, Germany) according to the manufacturer's instructions. Plates containing 200,000 cells were co-transfected with 0.5 µg of the reporter plasmid together with an expression plasmid containing the β-galactosidase gene-coding region (pSV40-βGAL). Cells were inoculated in 24-well plates and maintained for 48 h. These cells were harvested and lysed in reporter lysis buffer (Promega). Cell extracts were assayed for luciferase and β-galactosidase activity (β-Gal Reporter Gene Assay, Roche), which was used to normalize the results. All experiments were performed at least three times in duplicate well.

### Cell cultures and treatments

SH-SY5Y (human neuroblastoma, ATCC CRL-2266) cell line was maintained in low glucose Dulbecco's modified Eagle's medium (Sigma-Aldrich Inc., St. Louis, MO, USA), while HeLa (human cervix adenocarcinoma, ATCC CCL-2) and NT2 (human teratocarcinoma, ATCC CRL-1973) cell lines were cultured in high glucose Dulbecco's modified Eagle's medium (Sigma). All media were supplemented with 10% heat inactivated fetal bovine serum (Biochrom AG, Berlin, Germany), 2 mM L-glutamine (Sigma), 100 units/mL penicillin and 100 mg/mL streptomycin (Sigma), with the exception of NT2 culture medium that was supplemented with 4 mM L-glutamine. All cell cultures were carried out at 37°C in humidified 5% CO_2_.

Dose response assays of 5-Aza-2′-deoxycytidine (DAC) and Trichostatin A (TSA) were performed as stated in Milagre and collaborators (2008) [34]. Subsequently, cells were treated with 5 µM DAC and 0.25 µM TSA for the indicated periods (or vehicle as control) and every 24 hours the medium was changed.

### RNA isolation and qPCR for *PDHA2* expression analysis

Total RNA was prepared from all cells, treated and untreated, using the RNeasy® Mini Kit (Qiagen) and contamination by genomic DNA was eliminated from each RNA sample by pre-treatment with DNase I using RNase-free DNase Set (Qiagen).

Total RNA was reverse transcribed using the Reverse Transcription System A3500 Kit (Promega) with random primers following the manufacturer's instructions.

First strand DNA (1 µg) was used as template for quantitative real-time PCR with an ABI PRISM 7300 sequence detection system (Applied Biosystems). The cycling conditions were 95°C/10 min followed by 40 cycles of 95°C/15 sec and 60°C/1 min. It was used the TaqMan® Gene Expression Assay - ID: Hs01043024_s1 (Applied Biosystems) for specific detection of PDHA2 mRNA, and the TaqMan® β-actin Control Reagents as endogenous control (Applied Biosystems). In every reaction we used the TaqMan® Gene Expression Master Mix (Applied Biosystems) following enclosed instructions. Each sample was assayed in triplicate and results show a minimum of three independent experiments. Transcript levels were normalized to β-actin and expressed in pg *PDHA2* mRNA per ng of β-actin mRNA.

Additionally, we analyzed *PDHA2* mRNA by conventional RT-PCR analysis with a gene specific set of primers, designed to amplify the 5′ region of the transcript: the forward primer 5′-TGCCATCTACAGCACTCCGT-3′ hybridizing to nucleotides −27 to −8 and the reverse primer 5′-AGCACAACCTCCTCTTCTTCC-3′ hybridizing to nucleotides +412 to +432. The 459 pb product was amplified with SYBR green Master Mix in an ABI 7300 sequence detection system (Applied Biosystems) and visualized by agarose gel electrophoresis.

### Genomic DNA isolation and bisulfite sequencing for *PDHA2* methylation analysis

Genomic DNA was isolated from treated and untreated cells using a salting-out method (Citogene® DNA Blood Kit – Citomed, Lisbon, Portugal).

The number and distribution of scattered CpG sites and CpG islands of *PDHA2* gene has been previously analyzed [14] using the online program MethPrimer, available at www.urogene.org/methprimer, which defines CpG islands as sequences longer than 200 bp, with a calculated CG composition >50% and an observed to expected CpG ratio of >0.6 [35].

Bisulfite PCR sequencing was carried out using the EpiTect® Bisulfite Kit (Qiagen) and CpG islands were amplified by PCR using the specific primers and the conditions previously described [14].

The reaction products were purified by MinElute® PCR Purification Kit (Qiagen) and sequenced in both directions by primer walking with ABI Prism BigDye Terminator Cycle Sequencing Ready Reaction Kits, in an ABI PRISM 310 Genetic Analyzer (Applied Biosystems, Foster City, CA, USA).

### Chromatin immunoprecipitation assay

Chromatin immunoprecipitation assays were performed as described previously [36]. Briefly, chromatin isolated from cell cultures was immunoprecipitated using the following antibodies: anti RNA pol II clone CTD4H8 (#05-623, Millipore, Bedford, MA, USA), H3K36me3 (#ab9050, Abcam, Cambridge, UK), Sp1 (PEP 2) X (#SC-59X, Santa Cruz Biotechnology, Inc., Santa Cruz, CA, USA) and normal rabbit immunoglobulin (#X0903, DakoCytomation, Denmark). The recovered DNA was analyzed by quantitative real-time PCR with SYBR green Master Mix in an ABI 7300 sequence detection system (Applied Biosystems). The qPCRs were performed using primers designed to cover three different regions of the *PDHA2* gene: the proximal promoter region (+1 bp) 5′-GGCAGGCACTGTACAAATCA-3′ (forward) and 5′-CAGTGCACACGGGTGATAGA-3′ (reverse); the CpG island II region (+385 bp) 5′-GGGCTCATGGTGTGTGCTAT-3′ (forward) and 5′-AGCACAACCTCCTCTTCTTCC-3′(reverse); and a distal upstream region (−10 Kb) 5′-GCATGGCAGGACTTCTCTC-3′ (forward) and 5′-TTACAGGCAATGCTTG ACCA-3′ (reverse).

### Statistical analysis

Statistical analysis was performed using the Student's *t*-test and the ANOVA one-way test with the Tukey Honestly Significant Differences (HSD) *post-hoc* test, the Tukey HSD for unequal N (Spjotvoll/Stoline test). All analyses were performed using the STATISTICA (data analysis software system), version 7.1 StatSoft, Inc. (Tulsa, OK, USA; 2006). A value of p<0.05 was considered significant.

## Supporting Information

Table S1 **Statistical analysis of** ***PDHA2*** **gene methylation results by the ANOVA one-way test.**
(DOCX)
