# Supplementary material for: Demethylation of the Coding Region Triggers the Activation of the Human Testis-Specific PDHA2 Gene in Somatic Tissues
Source: PLoS One. 2012 Jun 1;7(6):e38076. doi: 10.1371/journal.pone.0038076 (PMC3365900; doi:10.1371/journal.pone.0038076)
Supplement: Table S1 — Statistical analysis of PDHA2 gene methylation results by the ANOVA one-way test. (DOCX) [file pone.0038076.s001.docx]

Table S1. Statistical analysis of *PDHA2* gene methylation results by the ANOVA one-way test.

| **CpG site^#^** | **F** | **df** | **p** |
| --- | --- | --- | --- |
| +276 | 3.596 | 3 | 0.050* |
| +297 | 1.145 | 3 | 0.371 |
| +312 | 4.683 | 3 | 0.015* |
| +326 | 6.498 | 3 | 0.004* |
| +333 | 9.594 | 3 | 0.001* |
| +373 | 20.64 | 3 | 0.000* |
| +388 | 8.203 | 3 | 0.001* |
| +399 | 20.73 | 3 | 0.000* |
| +410 | 19.5 | 3 | 0.000* |
| +449 | 5.181 | 3 | 0.011* |

*^#^ CpG Number in relation to ATG*

** Statistical significance p <0.05*
